# Analysis of polyunsaturated fatty acids in antipsychotic-free individuals with at-risk mental state and patients with first-episode schizophrenia

**DOI:** 10.3389/fpsyt.2023.1188452

**Published:** 2023-07-26

**Authors:** Anh Thi Phuong Le, Yuko Higuchi, Tomiki Sumiyoshi, Hiroko Itoh, Daiki Sasabayashi, Tsutomu Takahashi, Michio Suzuki

**Affiliations:** ^1^Department of Neuropsychiatry, University of Toyama Graduate School of Medicine and Pharmaceutical Sciences, Toyama, Japan; ^2^Research Center for Idling Brain Science, University of Toyama, Toyama, Japan; ^3^Department of Preventive Intervention for Psychiatric Disorders, National Institute of Mental Health, National Center of Neurology and Psychiatry, Tokyo, Japan; ^4^Department of Psychiatry, National Center of Neurology and Psychiatry Hospital, Tokyo, Japan

**Keywords:** polyunsaturated fatty acid, omega-3 polyunsaturated fatty acid, omega-6 polyunsaturated fatty acid, n-3 polyunsaturated fatty acid, n-6 polyunsaturated fatty acid, nervonic acid, at-risk mental state, first-episode schizophrenia

## Abstract

**Introduction:**

Abnormalities in membrane phospholipids are considered one of the pathophysiological backgrounds for schizophrenia. This study, explores the fatty acid composition of erythrocyte membranes and its association with clinical characteristics in two groups: individuals with an at-risk mental state (ARMS) and patients experiencing their first-episode of schizophrenia (FES).

**Materials and methods:**

This study measured erythrocyte membrane fatty acids in 72 antipsychotic-free individuals with ARMS, 18 antipsychotic-free patients with FES, and 39 healthy volunteers. Clinical symptoms and cognitive and social functions were assessed using the Positive and Negative Syndrome Scale (PANSS), Brief Assessment of Cognition in Schizophrenia (BACS), Schizophrenia Cognition Rating Scale (SCoRS), and Social and Occupational Functioning Assessment Scale (SOFAS).

**Results:**

Eicosapentaenoic and docosapentaenoic acid levels were lower in the ARMS and FES groups than in the healthy control group. In contrast, nervonic acid (NA) levels were markedly higher in the ARMS and FES groups than in the controls, while only the FES group showed higher levels of arachidonic acid. Oleic acid and NA levels were significantly associated with PANSS scores in both the FES and ARMS groups, particularly for the negative and general subscores. However, the patient groups had no significant associations between the fatty acid composition and the BACS, SCoRS, and SOFAS scores. Furthermore, the baseline fatty acid composition did not differ between the ARMS individuals who later developed psychosis (*N* = 6) and those who were followed for more than 2 years without developing psychosis onset (*N* = 30).

**Discussion:**

The findings suggest that abnormal fatty acid compositions may be shared in the early stages of schizophrenia and the clinical high-risk state for psychosis and may serve as vulnerability markers of psychopathology.

## 1. Introduction

Altered compositions of membrane phospholipids, which reflect the fluidity and elasticity of cell membranes (1, 2), have been implicated in the pathogenesis of schizophrenia (3–6). Specifically, decreased omega-3 (*n*-3) polyunsaturated fatty acid (PUFA) (7) in the erythrocyte membrane may reflect a core trait characteristic of the illness. Erythrocyte membrane PUFA level was estimated to better reflect neural cell membrane fatty acid composition than plasma PUFA level shown by several studies conducted on humans (8–10), and animal (11, 12). Decreased erythrocyte membrane PUFA levels exists even in the first-episode of schizophrenia (1, 6) and may also be associated with cognitive impairments (13, 14) and negative symptomatology (15, 16). However, the concentration of essential PUFAs, which cannot be synthesized *de novo* and require dietary intake (3, 5), is considerably affected by environmental factors such as physical condition, dietary habits, and antipsychotic medication (1, 7, 17). Furthermore, a few previous studies of erythrocyte membrane nervonic acid (NA), an n-9 monounsaturated fatty acid (FA) that is a major component of the myelin membrane (17), have reported both increased (18) and decreased (19) levels in medicated patients with schizophrenia. Thus, further comprehensive (i.e., saturated, monounsaturated, and PUFA) analyses in antipsychotic-free patients and well-controlled (e.g., physical condition, general biochemical data) comparison subjects are required to clarify the role of FA in the pathophysiology of schizophrenia.

Recently, studies have demonstrated reduced erythrocyte (20) or plasma (21) n-3 PUFAs in individuals with a clinical high-risk state for psychosis, known as the at-risk mental state (ARMS) (22, 23), of which approximately 30% develop psychosis within 2 years (24, 25). These findings suggest that deficits in PUFAs may be present prior to psychosis onset, serving as a potential trait marker. These findings may support the hypothesized relationship between PUFAs and early neurodevelopment (26), where low nutrition exposure during the brain’s neurodevelopmental period may cause epigenetic changes (27), leading to various neuropsychiatric disorders, including psychosis. Similar to schizophrenia, decreased membrane n-3 PUFAs seem to contribute to negative symptoms (28) and cognitive deficits (such as impaired verbal fluency) (29, 30) in individuals with ARMS who are less affected by antipsychotics. High-risk individuals may also be characterized by an increased proportion of NA (20), associated with a range of prodromal symptomatology (28, 31). However, conflicting results, such as normal n-3 PUFA [docosapentaenoic acid (DPA) and docosahexaenoic acid (DHA)] levels (32), have also been reported in individuals with ARMS. Furthermore, it remains largely unknown whether the FA findings in ARMS are associated with clinical outcomes, such as later psychosis onset. Amminger et al. (33) demonstrated that “decreased” NA but not PUFA levels predicted the future transition into psychosis in ARMS individuals. Therefore, FA composition in ARMS and its relationship with clinical characteristics, including symptoms, cognitive and social functions, and outcome, require further investigation compared to overt schizophrenia.

This study comprehensively measured erythrocyte membrane FAs in antipsychotic-free individuals with ARMS, antipsychotic-free patients with first-episode schizophrenia (FES), and healthy control subjects. The examined FAs included saturated FAs [palmitic acid (PA) and stearic acid (SA)], n-9 monounsaturated FAs [oleic acid (OA) and NA], n-3 PUFAs [eicosapentaenoic acid (EPA), DPA, and DHA], and n-6 PUFAs [linoleic acid (LA), dihomogammalinolenic acid (DGLA), and arachidonic acid (AA)]. The incorporation of these fatty acids was essential for a comprehensive analysis due to their significance as major constituents, comprising over 90% of the total fatty acid content in erythrocytes and the brain (1, 34). Based on previous findings, we predicted that both clinical groups would have an altered FA composition (especially a decreased n-3 PUFA and increased NA) and that such alterations would contribute to their symptom severity and cognitive functions. We also explored whether FA findings in the patients were associated with the illness stages of psychosis (ARMS vs. FES) and outcome of high-risk individuals (ARMS with vs. without later psychosis onset).

## 2. Materials and methods

### 2.1. Participants

Ninety Japanese patients from the University of Toyama Hospital participated in this study. They were diagnosed with either ARMS (34 males and 38 females; mean age ± standard deviation = 18.8 ± 4.4 years) or FES (11 males and 7 females; 24.5 ± 8.2 years). None of the patients took antipsychotic medications within 2 weeks of blood sampling; 61 of 72 ARMS patients and 11 out of 18 FES patients were antipsychotic-naïve. Thirty-nine healthy volunteers (21 males and 18 females; 28.9 ± 5.5 years) were recruited from university students, hospital staff, and acquaintances. Table 1 presents the demographic and clinical data.

**Table 1:** Demographic and clinical data for patients.

	H	ARMS	FES	Statistics	Group difference^a^
	*n* = 39	*n* = 72	*n* = 18		
Age (years)	28.9 (5.5)	18.9 (4.5)	24.5 (8.1)	*χ*^2^ = 56.6	*p* < 0.001**, H > FES > ARMS
Gender (female/male)	21/18	34/38	11/7	*χ*^2^ = 1.27	*p* = 0.52
Age at onset (years)	–	–	23.8 (8.3)	–	–
Duration of illness (years)			0.7 (0.4)	–	–
Socioeconomic status	6.6 (0.6)	3.6 (1.5)	4.4 (1.4)	*χ*^2^ = 34.7	*p* < 0.001**, H > ARMS, FES
Parental socioeconomic status	6.2 (0.8)	4.9 (0.9)	5.1 (0.9)	*χ*^2^ = 20.1	*p* < 0.001**, H > ARMS, FES
BMI (kg/m^2^)	20.1 (1.6)	21.1 (4.5)	22.4 (3.5)	*χ*^2^ = 3.8	*p* = 0.15
JART	–	96.5 (9.9)	99.6 (9.0)	U_70,17_ = 487.5	*p* = 0.25
PANSS					
: positive	–	12.0 (2.9)	17.1 (3.4)	U_70,16_ = 128.5	*p* < 0.001**, ARMS < FES
: negative	–	15.3 (6.4)	16.7 (6.1)	U_70,16_ = 473.5	*p* = 0.34
: general psychopathology	–	30.2 (7.8)	33.4 (7.0)	U_70,16_ = 418.0	*p* = 0.12
: total	–	57.5 (14.1)	67.3 (13.1)	U_70,16_ = 343.0	*p* = 0.02*, ARMS < FES
BACS^b^	–	−0.62 (0.91)	−1.37 (1.09)	U_72,17_ = 348.0	*p* = 0.006**, ARMS > FES
SCoRS^c^	–	5.5 (2.2)	6.9 (2.0)	U_70,16_ = 351.5	*p* = 0.02*, ARMS < FES
SOFAS^d^	–	48.5 (10.3)	44.6 (12.5)	U_57,16_ = 365.5	*p* = 0.23

Values represent mean (S.D.). ARMS, at-risk mental state (ARMS); BACS, brief assessment of cognition in schizophrenia; BMI, body mass index; FES, first-episode schizophrenia; H, healthy control; JART, Japanese Adult Reading Test; PANSS, positive and negative syndrome scale; SCoRS, schizophrenia cognition rating scale; SOFAS, social and occupational functioning assessment scale.

a Demographic differences between groups were examined by Kruskal–Wallis test (age), qui-square test (gender), or Mann–Whitney U test (others). **p < 0.01 and *p < 0.05.

b BACS composite score was calculated by averaging all *z*-scores of the six primary measures from the BACS.

c Data are ranging from 0 to 10, with larger numbers representing more worse functions.

d Data are ranging from 0 to 100. Generally, Healthy subjects generally have a scores ranging from 90 to 100.

Patients diagnosed with schizophrenia underwent diagnostic interviews using the Structured Clinical Interview for DSM-IV Axis I Disorders (SCID-I) Patient Edition (35). FES was defined as an illness duration of fewer than 2 years and a single psychotic episode, following previous studies (36, 37). Recent-onset patients who had multiple psychotic episodes within 2 years were excluded. ARMS individuals were identified using the Comprehensive Assessment of At-Risk Mental State (CAARMS) (23), with diagnoses performed by experienced psychiatrists. ARMS individuals were further sub-grouped based on clinical outcomes during the follow-up period, as described in previous reports (24). Conversion to psychosis was defined according to the “psychotic disorder criteria” in CAARMS: (i) hallucinations, unusual thoughts, and suspiciousness exceed defined severities, or delusion with strong conviction, or conceptual disorganization exceeds moderate level, (ii) frequency of symptoms is at least several times a week, and (iii) the episode is longer than 1 week (23). In the subgroup analyses of the ARMS individuals, 36 subjects were excluded due to an insufficient short follow-up period (<2 years). During the follow-up period, six ARMS subjects developed psychosis (ARMS-P), with five developing schizophrenia and one developing delusional disorder. Thirty participants who did not develop psychosis were defined as ARMS-non-psychosis (ARMS-NP). The transition rate was 16.7%.

The study collected information on clinical history through interviews with the participants, their families, or medical records. Physical examination and standard laboratory tests confirmed that participants were physically healthy. Exclusion criteria included a history of substance abuse or dependence, seizures, head injury, and an estimated premorbid IQ of less than 70 based on the Japanese Adult Reading Test (38). Additional criteria for healthy controls were; (i) no Axis I disorders based on the SCID-I Non-patient Edition (35), and (ii) no personal or family (within first-degree relatives) history of psychiatric disorders.

This study was conducted following the principles of the Declaration of Helsinki and was approved by the Committee on Medical Ethics of Toyama University (no. I2013006) on February 5, 2014. Written informed consent was obtained from all participants after a full explanation of the study’s purpose and procedures were provided. For participants under 20, written consent was also obtained from their parents or guardians.

### 2.2. Clinical assessment

Experienced psychiatrists or psychologists evaluated clinical symptoms, cognitive function, and social function using the Positive and Negative Syndrome Scale (PANSS) (39), Brief Assessment of Cognition in Schizophrenia (BACS) (40, 41), Schizophrenia Cognition Rating Scale (SCoRS) (42, 43), and the Social and Occupational Functioning Assessment Scale (SOFAS) (44). BACS composite scores were obtained by averaging the *z*-scores of the six subtests (41). Clinical assessments were performed on the same day as blood collection or within 2 weeks of blood collection.

### 2.3. FA analysis

Blood samples were collected from study participants between 08:30 and 10:00 after at least 2 hours of fasting for FA measurements and general blood and biochemical examinations (Supplementary material 1 for detailed results). Erythrocyte membrane FA levels were analyzed using gas chromatography based on an established method (14, 34, 45). Briefly, 1 mL of red blood cells obtained from the subjects was collected into a 15 mL screw cap vial. The vial received 4.0 mL of 0.6 N methanolic HCl containing 4 μL of 0.5% butyl hydroxytoluene (BHT) as an internal standard and was then sealed and incubated at 80°C for 2 hours. Methylated FAs were extracted twice with hexane, and the layers were separated by centrifugation in a swinging rotor at 3000 g for 15 min at room temperature. The hexane layer was carefully removed and collected in separate vials. The hexane extract was dried entirely by passing through argon and stored at −40°C until use. The methylated FAs were resuspended in 150 μL hexane, and aliquots (1 μL) were used for FA analysis with a Shimadzu gas chromatograph (Model GC-2010, Japan), using a capillary column of dimensions 30 m × 0.32 mm × 0.20 μm (Supelco, United States). A flame ionization detector was used with a column oven temperature of 160°C for 10 min, programmed at 10°C rise/min up to 175°C, and held at 220°C for 10 min. The injector and detector temperatures were set to 240°C and 275°C, respectively. The column was calibrated by injecting a standard FA mixture at approximately equal proportions. The peaks in the recorded data were identified based on the retention time of standard FAs run under identical conditions.

The FA data were categorized into four groups: (i) saturated FAs (PA, SA), (ii) n-9 series monounsaturated FAs (OA, NA), (iii) n-3 PUFAs (EPA, DPA, DHA), and (iv) n-6 series PUFAs (LA, DGLA, AA). FA levels were expressed as relative values measured as 100% of the 11 FAs, which included the 10 FAs mentioned above and BHT as an internal standard (1). We calculated the following parameters based on the previous literatures: (i) n-3 total (EPA + DPA + DHA), (ii) n-6 total (LA + DGLA + AA), (iii) n-6/n-3 ratio (AA/[EPA + DHA]) as an index to assess the inflammatory response (46, 47), and (iv) omega-3 index (EPA + DHA) as a potential index to predict vulnerability to several neuropsychiatric conditions and functional outcome of ARMS (28, 48, 49).

### 2.4. Statistical analysis

Statistical analyses were performed using Statistical Package for Social Sciences version 25 (SPSS Japan Inc.) and Jamovi Software^1^. As most demographic/clinical data (age, scores for PANSS subscales, BACS, SCoRS, and SOFAS) had skewed distributions, nonparametric Mann–Whitney U (for two-group comparisons) or Kruskal–Wallis (for three-group comparisons) tests were used to compare group differences. Similarly, nonparametric tests were employed for group differences in FA compositions, which were found to have non-normal distributions. Spearman’s rho with semi-partial correlation was used to calculate the correlation between FA composition and clinical data, with FA indices being controlled by age, because significant age differences among the groups and age significantly affected NA, EPA, and DPA in our data (data not shown). To correct for multiple comparisons, post-hoc Dwass–Steel–Critchlow–Fligner tests were used for group comparisons. For correlation analyses between FA composition and clinical variables, the Benjamini–Hochberg false discovery rate (FDR) procedure was used because there were many items to be compared (50). Significance was set at a value of *p* less than 0.05. In the cases of FDR-adjusted *p*-values, significance was set at less than 0.1 according to the previous literatures (51–53) where screening many items was done.

## 3. Results

### 3.1. Subjects’ profile

The gender ratios of the groups were matched, but there were significant differences in age (controls > FES > ARMS) and personal/parental socioeconomic status (Table 1). Body mass index did not differ between the groups. Japanese Adult Reading Test scores did not differ between the ARMS and FES groups. As expected, the FES group had lower BACS scores, higher SCoRS scores, and higher PANSS positive symptom scores than the ARMS group. The ARMS and FES groups had relatively high alkaline phosphatase levels, which fell within the normal range for adolescents (Supplementary material 1). Prolactin (PRL) levels were examined in 78 patients, of which 12 (10 males and 2 females, 15.4%) exceeded the normal range. Prolactin levels can be elevated even in antipsychotic-free schizophrenia patients without apparent physical illness (54, 55), so patients with high levels were not excluded from this study. Some of the patients were taking anxiolytics (15.6%), hypnotics (11.1%) and antidepressants (8.9%). However, these medications did not affect clinical or cognitive indices, or fatty acids composition (data not shown).

### 3.2. FA composition

**Figure 1:**
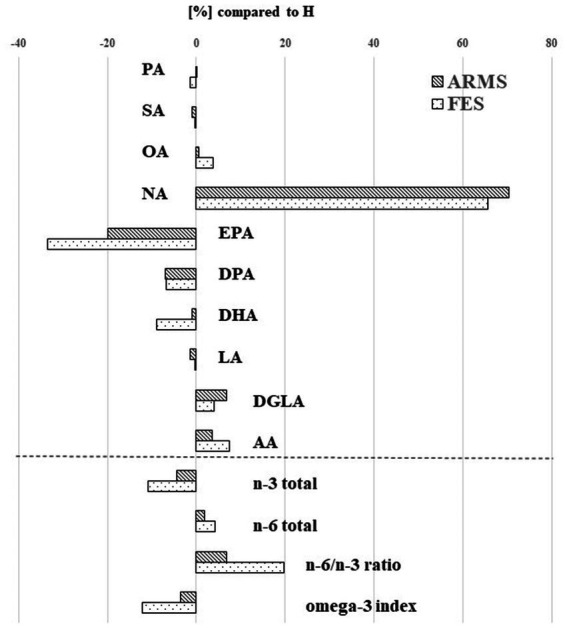
Percentage of fatty acid levels in the at-risk mental state (ARMS) and first-episode schizophrenia (FES) groups compared to the healthy control (H) group. AA, arachidonic acid (20:4 n-6); DGLA, dihomogammalinolenic acid (20:3 n-6); DHA, docosahexaenoic acid (22:6 n-3); DPA, docosapentaenoic acid (22:5 n-3); EPA, eicosapentaenoic acid (20:5 n-3); LA, linoleic acid (18:2 n-6); NA, nervonic acid (24:1 n-9); OA, oleic acid (18:1 n-9); PA, palmitic acid (16:0); SA, stearic acid (18:0).

Table 2 and Figure 1 present the results of FA composition analysis. EPA and DPA levels were significantly lower in the ARMS and FES groups than in healthy controls. The NA level was markedly higher in the ARMS and FES groups, while the AA level was significantly higher only in the FES group compared to the controls. Regarding summary values, the FES group had significantly lower n-3 total and higher n-6 total scores than the controls. These findings remained consistent even when we analyzed only antipsychotic-naïve FES/ARMS subjects (data not shown).

**Table 2:** Fatty acid composition.

		H	ARMS	FES	*χ* ^2^	*p*
		*n* = 39	*n* = 72	*n* = 18		
Saturated	PA	21.14 (0.99)	21.17 (0.98)	20.86 (1.08)	0.91	0.64
	SA	19.74 (0.65)	19.57 (0.75)	19.68 (0.88)	3.70	0.16
n-9 monounsaturated	OA	14.74 (1.08)	14.84 (1.11)	15.31 (1.19)	2.86	0.24
	NA	0.54 (0.07)	0.92 (0.29)	0.89 (0.29)	61.27	<0.0001***, H < ARMS, FES
n-3 polyunsaturated	EPA	1.30 (0.46)	1.04 (0.40)	0.87 (0.23)	14.92	0.0006***, H > ARMS, FES
	DPA	3.00 (0.31)	2.79 (0.27)	2.80 (0.25)	15.28	0.0005***, H > ARMS, FES
	DHA	8.29 (1.22)	8.22 (1.33)	7.56 (0.86)	5.99	0.05
n-6 polyunsaturated	LA	10.24 (0.97)	10.10 (1.03)	10.20 (1.12)	0.68	0.71
	DGLA	1.43 (0.19)	1.53 (0.26)	1.49 (0.30)	4.78	0.09
	AA	15.22 (1.43)	15.77 (1.13)	16.36 (1.57)	10.63	0.005**, H < FES
Summary value	n-3 total	12.60 (1.79)	12.05 (1.72)	11.22 (1.11)	9.65	0.008**, H > FES
	n-6 total	26.89 (1.72)	27.40 (1.24)	28.05 (1.20)	7.06	0.03*, H < FES
	n-6/n-3 ratio^a^	1.65 (0.46)	1.77 (0.41)	1.98 (0.38)	9.67	0.008**, H < FES
	Omega-3 Index^b^	9.59 (1.57)	9.26 (1.63)	8.42 (0.97)	8.62	0.01*, H > FES

Values represent mean (S.D.) of the percentage of the total fatty acids. AA, arachidonic acid (20:4 n-6); ARMS, at-risk mental state; DGLA, dihomogammalinolenic acid (20:3 n-6); DHA, docosahexaenoic acid (22:6 n-3); DPA, docosapentaenoic acid (22:5 n-3); EPA, eicosapentaenoic acid (20:5 n-3); FES, first-episode schizophrenia; H, healthy control; LA, linoleic acid (18:2 n-6); NA, nervonic acid (24:1 n-9); OA, oleic acid (18:1 n-9); PA, palmitic acid (16:0); SA, stearic acid (18:0). Differences between groups were examined by Kruscal–Wallis test with post-hoc comparisons. ***p < 0.001, **p < 0.01, and *p < 0.05.

a n-6/n-3 ratio = AA/(EPA + DHA).

b Omega-3 index = EPA + DHA.

### 3.3. Relationships between FA component and clinical variables

The correlation results are presented in Table 3; Supplementary material 2. A significant positive correlation was found between NA level and PANSS scores in the ARMS group, particularly for negative syndrome and general psychopathology scores. A significant positive correlation was found between OA level and total PANSS score in the FES group. No significant correlation was found between FA levels and BACS, SCoRS, or SOFAS scores.

**Table 3:** Relationships between fatty acid levels and clinical/cognitive indices.

		PANSS total		PANSS positive		PANSS negative		PANSS general psychopathology		BACS		SCoRS		SOFAS	
		ARMS	FES	ARMS	FES	ARMS	FES	ARMS	FES	ARMS	FES	ARMS	FES	ARMS	FES
Saturated	PA	0.18	0.05	0.03	−0.01	0.22	0.25	0.14	−0.16	0.02	−0.03	−0.14	0.20	−0.03	−0.64^†^
	SA	−0.14	−0.46	−0.13	−0.58^†^	−0.14	−0.37	−0.05	−0.34	−0.19	0.26	0.23	−0.13	−0.16	0.06
n-9 monounsaturated	OA	−0.12	0.71*	−0.07	0.25	−0.10	0.63^†^	−0.17	0.61^†^	0.11	−0.60^†^	0.11	0.08	−0.06	−0.14
	NA	0.43*	0.17	0.19	−0.27	0.36*	0.13	0.36*	0.33	−0.05	−0.17	−0.17	−0.09	0.20	0.19
n-3 polyunsaturated	EPA	0.04	−0.17	0.11	0.15	0.09	−0.18	−0.04	−0.17	0.03	−0.05	−0.22	0.12	0.25	−0.20
	DPA	0.12	0.03	0.18	−0.14	0.13	0.01	0.05	0.17	0.13	−0.39	−0.10	−0.20	0.08	0.25
	DHA	0.20	0.01	0.14	0.10	0.18	−0.05	0.15	0.15	0.02	−0.29	−0.20	0.27	0.24	0.21
n-6 polyunsaturated	LA	−0.19	0.04	−0.05	0.54^†^	−0.22	−0.28	−0.17	0.16	0.01	0.15	0.11	−0.27	−0.10	0.55^†^
	DGLA	0.08	0.07	0.09	0.38	0.00	−0.11	0.07	0.13	−0.21	−0.04	0.10	−0.14	−0.13	0.43
	AA	−0.05	−0.21	−0.04	−0.40	−0.04	−0.16	0.00	−0.18	0.03	0.19	−0.07	−0.03	−0.08	−0.23
summary value	n-3 total	0.18	−0.08	0.17	−0.09	0.18	−0.06	0.11	0.07	0.04	−0.32	−0.22	0.22	0.25	0.25
	n-6 total	−0.17	−0.07	−0.03	0.29	−0.23	−0.30	−0.09	−0.02	−0.04	0.54^†^	0.12	−0.38	−0.24	0.13
	n-6/n-3 ratio^a^	−0.16	−0.10	−0.13	−0.27	−0.16	−0.05	−0.09	−0.12	−0.02	0.29	0.18	−0.29	−0.22	−0.13
	omega-3 index^b^	0.17	−0.01	0.15	0.06	0.18	−0.01	0.11	0.09	0.01	−0.26	−0.22	0.33	0.25	0.10

Values are Spearman’s rho, calculated using semi-partial correlation analysis that only fatty acid indices were controlled by age as a covariate. *False discovery rate adjustment *p* value < 0.1 (written in bold letters). ^†^There was a significant correlation between fatty acid composition and clinical/cognitive indices, however, they did not survive after post-hoc analysis for multiple comparison. AA, arachidonic acid (20:4 n-6); ARMS, at-risk mental state; BACS, brief assessment of cognition in schizophrenia; DGLA, dihomogammalinolenic acid (20:3 n-6); DHA, docosahexaenoic acid (22:6 n-3); DPA, docosapentaenoic acid (22:5 n-3); EPA, eicosapentaenoic acid (20:5 n-3); FES, first-episode schizophrenia; H, healthy control; LA, linoleic acid (18:2 n-6); NA, nervonic acid (24:1 n-9); OA, oleic acid (18:1 n-9); PA, palmitic acid (16:0); PANSS, positive and negative syndrome scale; SA, stearic acid (18:0); SCoRS, schizophrenia cognition rating scale; SOFAS, social and occupational functioning assessment scale.

a n-6/n-3 ratio = AA/(EPA + DHA).

b omega-3 index = EPA + DHA.

### 3.4. FA levels and diagnostic outcome of ARMS

Baseline FA levels did not differ significantly between the ARMS-P (*N* = 6) and ARMS-NP (*N* = 30) subgroups (Supplementary material 3).

### 3.5. Potential role of antipsychotics and illness chronicity on FA levels

We compared the FA levels between the current antipsychotic-free patients and an independent cohort receiving antipsychotics (Supplementary material 4). The medicated group had significantly higher n-3 PUFA levels in the ARMS and FES groups (Supplementary material 5). Correlation analyses showed a strong effect of antipsychotics on FA composition in the FES group but not in the ARMS group (Supplementary material 6). Furthermore, illness duration did not correlate with FA composition in the FES group (Supplementary material 7).

## 4. Discussion

To our knowledge, this is the first comprehensive study to investigate the erythrocyte membrane FA composition in antipsychotic-free patients with both ARMS and FES in comparison with healthy controls, as well as its relationship with symptom severity, social and cognitive functions assessed by the BACS, SOFAS, and SCoRS, and other clinical characteristics. Our findings showed that both clinical groups had decreased n-3 PUFAs (EPA and DPA) and increased NA, an n-9 monounsaturated FA, compared to the controls, regardless of the outcome of the ARMS group. We also found that n-9 monounsaturated FA (OA and NA) levels were predominantly associated with symptoms in the FES and ARMS groups, while the FA composition was not significantly related to their social and cognitive functions. These results suggest that the ARMS and FES groups may share FA abnormalities as a potential vulnerability factor, which could contribute to symptomatology.

### 4.1. n-3 PUFAs

These findings of decreased EPA and DPA levels in the current ARMS and FES groups are consistent with previous research in FES (6, 7, 34) and ARMS (20, 32), indicating that these changes may be a trait characteristic in the early stages of psychosis and not solely explained by antipsychotic medication (1, 15) or other environmental factors such as smoking or dietary intake after the onset of psychosis (56). While the exact role of membrane PUFAs in the pathophysiology of psychosis remains unclear, animal and experimental studies have suggested that PUFA abnormalities can affect membrane properties in the central nervous system (e.g., fluidity, elasticity, and thickness) (2) and dopaminergic transmission (57). Our findings may also support the animal vulnerability model of psychosis (27). PUFA deficiency during early neurodevelopmental stages could cause epigenetic changes, such as DNA methylation, which affect the expression of developmentally regulated genes (58) and increase the risk of psychosis in adulthood.

### 4.2. n-6 PUFAs

We observed increased levels of n-6 PUFA, particularly AA, in the FES group compared to controls. Previous studies have reported increased (18) and decreased (1, 6, 20) n-6 PUFA in the early stages of psychosis. The reasons for this discrepancy are unclear, but the FA composition of erythrocyte membranes is influenced by various factors, including dietary FA composition, age, ethnicity, physical condition, genes, and gene-by-diet interactions (59). Nonetheless, these studies consistently found an increased n-6/n-3 ratio in schizophrenia (1, 6, 18) and ARMS (20) groups. n-6 PUFAs have a high turnover rate and compete with n-3 PUFAs through the same enzyme (60). Because AA-derived eicosanoids have more prominent inflammatory activity than n-3 PUFAs, the imbalance between n-6 and n-3 PUFAs, indicated by the increased n-6/n-3 ratio, may cause neuroinflammatory pathology in neuropsychiatric disorders (58, 59). Although the increase in the n-6/n-3 ratio was prominent in the FES group, it did not reach statistical significance in the ARMS group (Table 2). This may be due to that ARMS is not as severe as schizophrenia in terms of neuroinflammation as mentioned above. Future longitudinal studies, especially before and after the onset of psychosis in ARMS patients, must investigate whether the putative n-3/n-6 imbalance progresses during psychosis.

### 4.3. n-9 monounsaturated FAs

The most robust finding of this study was the increased level of n-9 monounsaturated FAs (especially NA) and its relationship with PANSS negative and general subscale scores in both the ARMS and FES groups. Increased levels of OA were also associated with the severity of cognitive deficits in the FES group (rho = −0.60), although this did not survive multiple comparison corrections. Since NA is abundant in brain white matter and plays a crucial role in myelin maturation and integrity (17), our results may be partly in line with neuroimaging evidence that abnormalities in brain connectivity contribute to trait characteristics of psychosis, such as negative symptomatology (61) and cognitive deficits (62) in ARMS (63) and schizophrenia (64). Previous studies on FAs in ARMS and FES have also demonstrated an increased NA level (18, 20) and its relationship with symptom severity (18, 28, 31). However, some of them showed the association of NA level also with positive symptomatology (28, 31). No statistically significant correlation between FA levels and positive symptoms in this study might be attributable to relatively low scores of the positive subscales in our sample. Conflicting results have also shown that lower NA levels contributed to impaired white matter integrity and severe negative symptoms in recent-onset psychosis (46). Therefore, these findings on n-9 monounsaturated FAs require replication in combination with imaging studies of brain connectivity.

### 4.4. Diagnostic outcome and PUFAs in ARMS

This study did not observe any significant differences in FAs between the ARMS-P (*N* = 6) and -NP (*N* = 30) groups at baseline, indicating that the erythrocyte membrane FA composition may reflect general vulnerability to psychopathology but does not predict future onset of psychosis. However, this study may not have had sufficient statistical power due to small sample size of ARMS-P subjects. In contrast, Amminger et al. (33, 47) reported that lower NA and n-3 PUFAs may predict psychosis in high-risk individuals (*N* = 40, transition rate = 28%). However, the relationship between NA and psychosis risk is complex, as ARMS and psychosis patients, including Amminger’s own ARMS cohort (20), are generally reported to have increased NA levels compared to healthy controls. The most recent study by Amminger et al. (65) that examined EPA, DHA and omega-3 index found no significant predictor for transition at both month 6 and 12 (65). Given the relatively small sample size of this study and previous studies, as well as the potential influence of various factors on both FA and transition rate, including FA supplementation (66, 67) and antipsychotic medication (1, 68), the potential use of FAs as a predictive marker for psychosis remains unclear and requires further investigation.

### 4.5. Limitations

This study has several limitations. First, the small sample size, especially for the FES and ARMS-P groups, may have limited the statistic power of our results. Additionally, there was a significant age difference between groups (control > FES > ARMS), which we statistically controlled for in our analyses. However, future studies with larger age-matched samples must confirm our findings. Second, we did not control for the dietary habits of our participants. Although all participants had standard body mass indices (Table 1) and laboratory data (Supplementary material 1), environmental factors such as dietary habits may have influenced our FA results. However, one of the strengths of the study may be that there were no race differences, which is considered a limiting factor in other international collaborative studies (20). Third, as our study is cross-sectional, future longitudinal studies are needed to confirm the role of FA changes as a trait marker and to investigate the influence of illness stages. Fourth, as FA abnormalities have been reported in other neuropsychiatric disorders such as major depression (69), further research is necessary to confirm our findings’ disease specificity and investigate the potential influence of comorbid anxiety/depressive symptoms in patients with ARMS. Fifth, we failed to investigate the duration of symptom of ARMS that might have affected the results. Lastly, tobacco use should have been checked, however, we lacked this information.

### 4.6. Conclusion

This study found that the ARMS and FES groups exhibited similar FA abnormalities, including decreased n-3 PUFAs (EPA and DPA) and increased n-9 monounsaturated FA (NA) levels, regardless of previous antipsychotic exposure. Additionally, we found that the altered n-9 monounsaturated FA levels were associated with symptoms, measured by PANSS especially negative symptom and general psychopathology but not social or cognitive functions in the early stages of psychosis. Our findings support the notion that an altered composition of membrane phospholipids may be a characteristic of psychosis. We observed no significant influence of illness stages or outcomes of high-risk individuals on the FA composition. However, the potential for FA changes during psychosis and the neural substrates associated with these findings should be examined in future longitudinal studies that employ neuroimaging methods.

## Data availability statement

The raw data supporting the conclusions of this article will be made available by the authors, without undue reservation.

## Ethics statement

The studies involving human participants were reviewed and approved by the Committee on Medical Ethics of the University of Toyama. Written informed consent to participate in this study was provided by the participants’ legal guardian/next of kin.

## Author contributions

TS, MS, TT, YH, and AL conceived the idea and design of this study. TS, YH, TT, DS, and MS recruited subjects and were involved in the clinical assessments. HI was used to measure the fatty acid components. YH, DS, and AL were involved in data collection. YH, AL, and DS were responsible for entering data and data analyses. MS, YH, and AL interpreted the results. AL wrote the manuscript. MS, TS, TT, and YH contributed to the writing, checking, and editing of the manuscript. All authors contributed to the article and approved the submitted version.

## Funding

This study was supported by the Japan Society for the Promotion of Science KAKENHI (grant numbers 16K10205, 18K07550, 20H03598, 26461739, and 22K07554), and the Japan Agency for Medical Research and Development (grant number JP19dk0307029) and the 47th SEISHIN Medical Research Foundation (2014) and the 10th Research Group for Schizophrenia (2014). The funding sources were not involved in the study design; data collection; data analyses; interpretation of results; writing of the report; or the decision to submit the article for publication.

## Conflict of interest

The authors declare that the research was conducted in the absence of any commercial or financial relationships that could be construed as a potential conflict of interest.

## Publisher’s note

All claims expressed in this article are solely those of the authors and do not necessarily represent those of their affiliated organizations, or those of the publisher, the editors and the reviewers. Any product that may be evaluated in this article, or claim that may be made by its manufacturer, is not guaranteed or endorsed by the publisher.

## Footnotes

^1^ https://www.jamovi.org
