# Supplementary material for: Analysis of polyunsaturated fatty acids in antipsychotic-free individuals with at-risk mental state and patients with first-episode schizophrenia
Source: Front Psychiatry. 2023 Jul 26;14:1188452. doi: 10.3389/fpsyt.2023.1188452 (PMC10410072; doi:10.3389/fpsyt.2023.1188452)
Supplement: Supplementary file 1 [file Data_Sheet_1.pdf]

Suppl 1. Laboratory Data

		normal range		ARMS		FES		t	df	p
		lower	upper	n=72		n=18				
TP	[g/dL]	6.6	8.1	7.4	(0.4)	7.2	(0.3)	1.47	85	0.15
Alb	[g/dL]	4.1	5.1	4.6	(0.3)	4.5	(0.2)	1.45	87	0.15
AMY	[U/L]	44	132	79.8	(25.2)	76.0	(25.7)	0.56	72	0.58
CK	[U/L]	59	248	111.0	(121.5)	72.7	(33.1)	1.32	87	0.19
GOT	[U/L]	13	30	19.8	(6.9)	22.7	(27.2)	-0.81	87	0.42
GPT	[U/L]	10	42	21.1	(19.8)	31.7	(66.4)	-1.18	87	0.24
LD	[U/L]	124	222	156.5	(25.2)	153.9	(24.3)	0.40	87	0.69
ALP	[U/L]	38	113	269.9	(161.7)	186.4	(105.5)	2.08	87	0.04*
γGTP	[U/L]	13	64	18.2	(11.5)	21.8	(20.7)	-0.99	87	0.33
BUN	[mg/dL]	8.0	20.0	11.3	(2.6)	11.5	(3.0)	-0.21	87	0.83
Cre	[mg/dL]	0.65	1.07	0.7	(0.1)	0.7	(0.1)	-0.58	87	0.57
UA	[mg/dL]	3.7	7.8	5.5	(1.4)	5.2	(1.3)	0.86	84	0.39
TG	[mg/dL]	40	149	87.3	(51.0)	92.7	(58.4)	-0.38	82	0.71
T-chol	[mg/dL]	142	219	176.0	(35.8)	173.9	(30.0)	0.22	84	0.82
HDL-C	[mg/dL]	40	90	57.0	(12.5)	55.7	(13.0)	0.39	82	0.70
LDL-C	[mg/dL]	65	139	104.6	(31.4)	103.2	(20.2)	0.18	82	0.86
Na	[mmol/L]	138	145	139.4	(1.6)	139.3	(1.1)	0.36	87	0.72
K	[mmol/L]	3.6	4.8	4.2	(0.4)	4.2	(0.3)	0.55	87	0.58
Cl	[mmol/L]	101	108	103.7	(1.3)	104.1	(1.1)	-1.15	87	0.25
Ca	[mg/dL]	8.8	10.1	9.6	(0.3)	9.4	(0.4)	2.03	76	0.05
P	[mg/dL]	2.7	4.6	3.9	(0.5)	3.6	(0.7)	1.76	73	0.08
Mg	[mg/dL]	1.6	2.6	2.0	(0.1)	2.0	(0.1)	-1.49	72	0.14
T-bil	[mg/dL]	0.4	1.5	0.8	(0.5)	0.7	(0.2)	1.07	87	0.29
D-bil	[mg/dL]	0.0	0.4	0.2	(0.1)	0.2	(0.1)	0.07	87	0.95
WBC	[x10^2/μL]	33.0	86.0	61.5	(16.4)	57.5	(17.1)	0.93	87	0.36
RBC	[x10^4/μL]	435	555	493.1	(48.2)	489.9	(44.6)	0.25	87	0.80
Hb	[g/dL]	13.7	16.8	14.4	(1.7)	14.1	(1.9)	0.77	87	0.44
Ht	[%]	40.7	50.1	42.8	(4.5)	42.1	(5.0)	0.59	87	0.55
MCV	[fL]	83.6	98.2	86.8	(3.8)	85.8	(4.7)	0.97	87	0.33
MCH	[pg]	27.5	33.2	29.3	(1.6)	28.7	(2.2)	1.27	87	0.21
MCHC	[g/dL]	31.7	35.3	33.7	(1.0)	33.4	(1.2)	1.11	87	0.27
Plt	[x10^4/μL]	15.8	34.8	25.3	(5.1)	24.5	(5.7)	0.58	87	0.57
Glu	[mg/dL]	73	109	92.8	(7.2)	95.8	(9.1)	-1.52	85	0.13
HbA1c	[%]	4.9	6.0	5.3	(0.3)	5.4	(0.3)	-1.96	82	0.05
TSH	[mIU/L]	0.61	4.23	2.0	(1.2)	1.7	(1.3)	1.03	74	0.30
freeT3	[pg/mL]	2.3	4.0	3.5	(0.5)	3.2	(0.4)	1.77	74	0.08
freeT4	[ng/dL]	0.9	1.7	1.4	(0.2)	1.3	(0.2)	1.10	74	0.27
PRL(male)	[ng/mL]	4.3	13.7	11.3	(5.8)	14.8	(7.2)	-1.32	35	0.20
PRL(female)	[ng/mL]	6.1	30.5	15.3	(7.4)	18.8	(13.3)	-1.05	39	0.30

Values represent mean (S.D.).

Differences between groups were examined by student's t-test (*$p < 0.05$).

"Abbreviate: Alb, albumin; ALP, alkaline phosphatase; AMY, amylase; ARMS, at-risk mental state; BUN, blood urea nitrogen; Ca, calcium; CK, creatine kinase; Cl, chloride; Cre, creatinine; D-bil, direct bilirubin; FES, first episode schizophrenia; free T4, thyroxine; freeT3, tri-iodothyronine; γGTP, gamma-glutamyl transferase; Glu, glucose; GOT, glutamic-oxaloacetic transaminase; GPT, glutamic-pyruvic transaminase; Hb, hemoglobin; HbA1c, hemoglobin A1c; HDL-C, high-density lipoprotein; Ht, hematocrit; K, potassium; LD, lactate dehydrogenase; LDL-C, low-density lipoprotein cholesterol; MCH, mean corpuscular hemoglobin; MCHC, mean corpuscular hemoglobin concentration; MCV, mean corpuscular volume; Mg, magnesium; Na, sodium; P, phosphate Plt, platelet; PRL, prolactin; RBC, red blood cell; T-bil, total bilirubin; T-chol, total cholesterol; TG, triglyceride; TP, total protein; TSH, thyroid stimulating hormone; UA, uric acid; WBC, white blood cell.

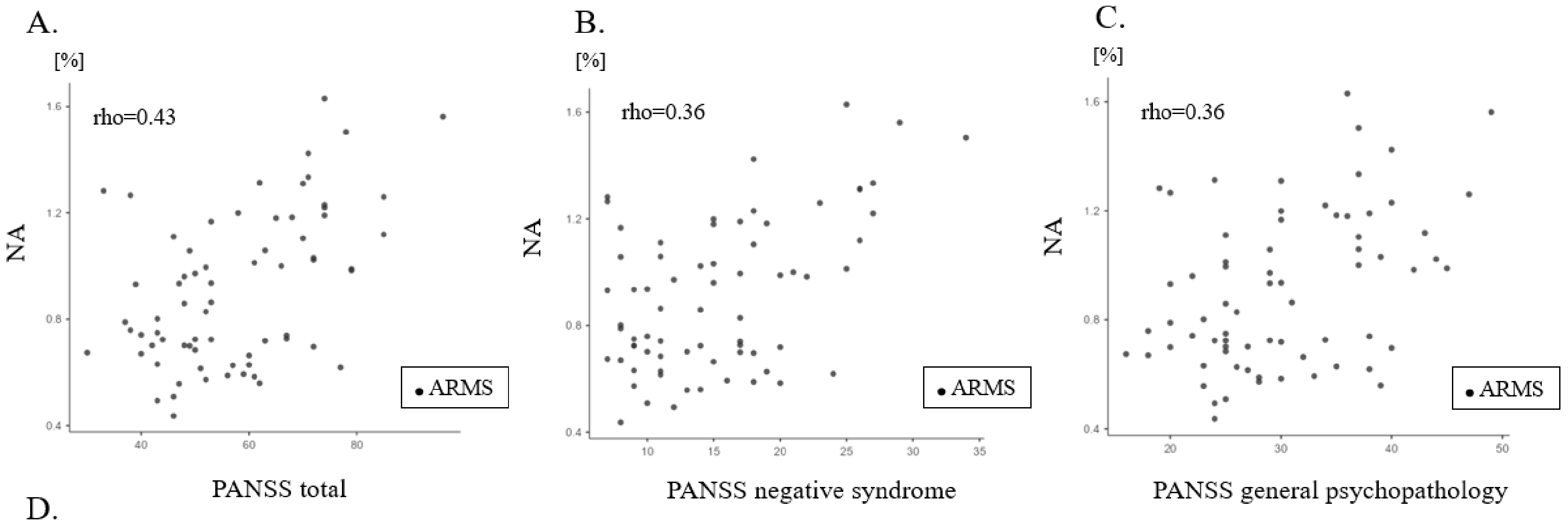

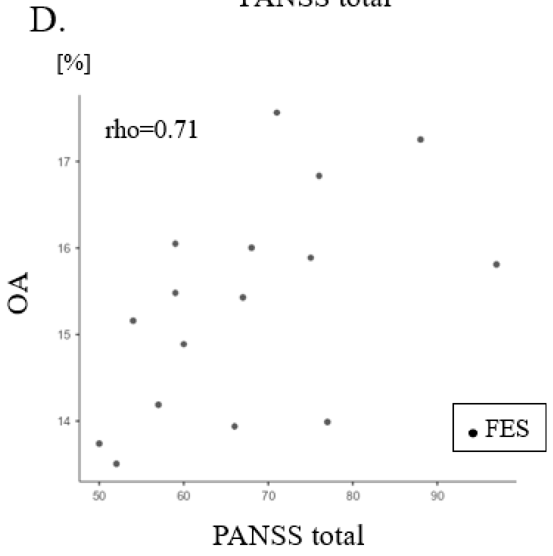

Suppl 2 The relationships between NA and PANSS scores

Note:
The relationships between NA and PANSS total score (A.), PANSS negative syndrome score (B.) and PANSS general psychopathology (C.) in ARMS.
The relationships between OA and PANSS total score (D.) in FES.

Spearman's rho was calculated between fatty acid concentrations and clinical data with semi-partial correlation, with fatty acid indices were controlled by age.

Abbreviate: ARMS, at-risk mental state; FES, first episode schizophrenia; NA, nervonic acid (24:1 n-9); OA, oleic acid (18:1 n-9); PANSS, Positive and Negative Syndrome Scale.

Suppl 3. Demographic, clinical and FAs data for *ARMS-P and ARMS-NP*

		ARMS-NP	ARMS-P	Statistics	Group difference[a]
		n=30	n=6		
Age [years]		18.4 (4.1)	19.2 (6.0)	$\chi2$=89.0	p=0.98
Gender (female/male)		9/21	3/3	$\chi2$=0.9	p=0.34
JART		95.4 (8.5)	91.7 (13.4)	$U_{30,6}$=78.0	p=0.62
PANSS					
:positive symptoms		12.1 (3.1)	14.5 (2.0)	$U_{29,6}$=51.5	p=0.12
:negative symptoms		17.0 (7.8)	16.7 (4.9)	$U_{29,6}$=85.5	p=0.97
:general psychopathology		31.1 (7.8)	36.7 (7.1)	$U_{29,6}$=51.0	p=0.12
:total		60.2 (15.3)	67.8 (12.8)	$U_{29,6}$=58.0	p=0.21
BACS[b]		-0.65 (0.81)	-1.46 (1.46)	$U_{30,6}$=57.0	p=0.17
SCoRS[c]		5.2 (2.4)	8.0 (0.9)	$U_{29,6}$=31.0	p=0.01*
SOFAS[d]		48.5 (9.8)	45.2 (10.1)	$U_{22,5}$=43.5	p=0.49
saturated	PA	21.40 (0.95)	21.07 (0.65)	$U_{30,6}$=77.0	p=0.61
	SA	19.41 (0.71)	19.39 (0.27)	$U_{30,6}$=79.0	p=0.66
n-9 monounsaturated	OA	14.85 (1.01)	15.16 (1.35)	$U_{30,6}$=80.0	p=0.69
	NA	1.00 (0.27)	1.01 (0.21)	$U_{30,6}$=84.0	p=0.82
n-3 polyunsaturated	EPA	1.09 (0.45)	1.01 (0.42)	$U_{30,6}$=85.0	p=0.85
	DPA	2.81 (0.29)	2.82 (0.15)	$U_{30,6}$=88.0	p=0.95
	DHA	8.41 (1.01)	8.62 (2.04)	$U_{30,6}$=87.0	p=0.92
n-6 polyunsaturated	LA	10.24 (0.95)	10.40 (1.51)	$U_{30,6}$=87.0	p=0.92
	DGLA	1.57 (0.27)	1.65 (0.43)	$U_{30,6}$=81.0	p=0.73
	AA	15.34 (1.01)	15.10 (0.66)	$U_{30,6}$=63.0	p=0.27
summary value	n-3 total	12.31 (1.50)	12.45 (2.38)	$U_{30,6}$=88.0	p=0.95
	n-6 total	27.15 (1.11)	27.14 (1.51)	$U_{30,6}$=89.0	p=0.98
	n-6/n-3 ratio[e]	1.65 (0.28)	1.64 (0.36)	$U_{30,6}$=87.0	p=0.92
	omega-3 index[f]	9.50 (1.35)	9.63 (2.32)	$U_{30,6}$=89.0	p=0.98

Note: Values represent mean (S.D.). Fatty acid values are shown as the percent of the total fatty acids. Abbreviate: AA, arachidonic acid (20:4 n-6); ARMS-NP, at-risk mental state non-psychosis; ARMS-P, at-risk mental state psychosis; BACS, Brief Assessment of Cognition in Schizophrenia; DGLA, dihomogammalinolenic acid (20:3 n-6); DHA, docosahexaenoic acid (22:6 n-3); DPA, docosapentaenoic acid (22:5 n-3); EPA, eicosapentaenoic acid (20:5 n-3); FES, first episode schizophrenia; H, healthy control; JART, Japanese Adult Reading Test; LA, linoleic acid (18:2 n-6); NA, nervonic acid (24:1 n-9); OA, oleic acid (18:1 n-9); PA, palmitic acid (16:0); PANSS, Positive and Negative Syndrome Scale; SA, stearic acid (18:0); SCoRS, Schizophrenia Cognition Rating Scale; SOFAS, Social and Occupational Functioning Assessment Scale.

a. Differences between groups were examined by qui-square test (gender) or Mann-Whitney U test (others) (* $p < 0.05$).

b. BACS composite score was calculated by averaging all z-scores of the six primary measures from the BACS.

c. Data are ranging from 0 to 10, with larger number representing more worse function.

d. Data are ranging from 0 to 100. Healthy subjects generally have a score ranging from 90 to 100.

e. n-6/n-3 ratio=AA/(EPA+DHA)

f. Omega3 index=EPA+DHA

Suppl 4. Demographic, clinical and FAs data for ***antipsychotic (+) and (-) subjects.***

		H	ARMS (n=93)				FES (n=55)			
		n=39	Antipsychotic(-), n=72	Antipsychotic (+), n=21	Statistic, (+) vs (-)[a]	p	Antipsychotic(-), n=18	Antipsychotic (+), n=37	Statistic, (+) vs (-)[a]	p
Age [years]		28.9 (5.5)	18.9 (4.5)	18.6 (3.0)	$U_{72,21}$=706	0.65	24.5 (8.1)	24.8 (7.4)	$U_{18,37}$=315	0.76
Gender (female/male)		21/18	34/38	11/10	$\chi2$=0.17	0.68	11/7	22/15	$\chi2$=0.01	0.90
Antipsychotic dosage (mg/day, resperidone		-	-	1.3 (1.0)	-	-	-	5.5 (4.9)	-	-
JART		-	96.5 (9.9)	99.3 (7.8)	$U_{70,19}$=569	0.34	99.6 (9.0)	99.8 (9.3)	$U_{17,36}$=298	0.87
PANSS		-								
:positive symptoms		-	12.0 (2.9)	12.6 (4.5)	$U_{70,20}$=673	0.80	17.1 (3.4)	14.4 (6.1)	$U_{16,36}$=185	0.04*
:negative symptoms		-	15.3 (6.4)	14.5 (5.6)	$U_{70,20}$=673	0.79	16.7 (6.1)	17.8 (18.0)	$U_{16,36}$=244	0.38
:general psychopathology		-	30.2 (7.8)	32.4 (7.7)	$U_{20,70}$=588	0.28	33.4 (7.0)	33.5 (8.7)	$U_{16,36}$=273	0.77
:total		-	57.5 (14.1)	59.5 (16.2)	$U_{70,20}$=649	0.62	67.3 (13.1)	65.8 (16.5)	$U_{16,36}$=254	0.51
BACS[b]		-	-0.62 (0.91)	-0.82 (0.84)	$U_{72,21}$=645	0.31	-1.37 (1.09)	-1.22 (0.73)	$U_{17,37}$=307	0.90
SCoRS[c]		-	5.5 (2.2)	4.4 (2.5)	$U_{70,19}$=485	0.07	6.9 (2.0)	5.1 (2.3)	$U_{16,37}$=169	0.01*
SOFAS[d]		-	48.5 (10.3)	48.8 (10.7)	$U_{57,13}$=366	0.95	44.6 (12.5)	41.6 (10.4)	$U_{16,28}$=195	0.48
saturated	PA	21.14 (0.99)	21.17 (0.98)	21.28 (1.04)	$U_{72,21}$=722	0.75	20.86 (1.08)	21.30 (0.88)	$U_{18,37}$=261	0.20
	SA	19.74 (0.65)	19.57 (0.75)	19.34 (0.99)	$U_{72,21}$=661	0.39	19.68 (0.88)	19.47 (0.71)	$U_{18,37}$=248	0.13
n-9 monounsaturated	OA	14.74 (1.08)	14.84 (1.11)	14.41 (1.18)	$U_{72,21}$=611	0.18	15.31 (1.19)	14.13 (1.13)	$U_{18,37}$=168	0.003**
	NA	0.54 (0.07)	0.92 (0.29)	1.05 (0.32)	$U_{72,21}$=563	0.08	0.89 (0.29)	0.99 (0.32)	$U_{18,37}$=272	0.28
n-3 polyunsaturated	EPA	1.30 (0.46)	1.04 (0.40)	1.31 (0.74)	$U_{72,21}$=614	0.19	0.87 (0.23)	1.48 (0.49)	$U_{18,37}$=73	<0.001***
	DPA	3.00 (0.31)	2.79 (0.27)	2.99 (0.30)	$U_{72,21}$=471	0.009**	2.80 (0.25)	3.03 (0.27)	$U_{18,37}$=174	0.005**
	DHA	8.29 (1.22)	8.22 (1.33)	8.58 (1.18)	$U_{72,21}$=644	0.30	7.56 (0.86)	8.83 (1.11)	$U_{18,37}$=117	<0.001***
n-6 polyunsaturated	LA	10.24 (0.97)	10.10 (1.03)	9.95 (1.44)	$U_{72,21}$=699	0.60	10.20 (1.12)	9.73 (1.11)	$U_{18,37}$=269	0.26
	DGLA	1.43 (0.19)	1.53 (0.26)	1.50 (0.26)	$U_{72,21}$=727	0.79	1.49 (0.30)	1.53 (0.31)	$U_{18,37}$=313	0.73
	AA	15.22 (1.43)	15.77 (1.13)	15.74 (1.16)	$U_{72,21}$=743	0.91	16.36 (1.57)	15.37 (1.19)	$U_{18,37}$=182	0.006**
summary value	n-3 total	12.60 (1.79)	12.05 (1.72)	12.87 (1.99)	$U_{72,21}$=603	0.16	11.22 (1.11)	13.34 (1.58)	$U_{18,37}$=90	<0.001***
	n-6 total	26.89 (1.72)	27.40 (1.24)	27.20 (2.05)	$U_{72,21}$=721	0.75	28.05 (1.20)	26.64 (1.43)	$U_{18,37}$=149	<0.001***
	n-6/n-3 ratio[e]	1.65 (0.46)	1.77 (0.41)	1.65 (0.34)	$U_{72,21}$=674	0.45	1.98 (0.38)	1.52 (0.28)	$U_{18,37}$=106	<0.001***
	omega-3 index[f]	9.59 (1.57)	9.26 (1.63)	9.89 (1.77)	$U_{72,21}$=635	0.27	8.42 (0.97)	10.31 (1.44)	$U_{18,37}$=91	<0.001***

Note: Values represent mean (S.D.). Fatty acid values are shown as the percent of the total fatty acids.
Abbreviate: AA, arachidonic acid (20:4 n-6); ARMS, at-risk mental state; BACS, Brief Assessment of Cognition in Schizophrenia; DGLA, dihomogammalinolenic acid (20:3 n-6); DHA, docosahexaenoic acid (22:6 n-3); DPA, docosapentaenoic acid (22:5 n-3); EPA, eicosapentaenoic acid (20:5 n-3); FES, first episode schizophrenia; H, healthy control; JART, Japanese Adult Reading Test; LA, linoleic acid (18:2 n-6); NA, nervonic acid (24:1 n-9); OA, oleic acid (18:1 n-9); PA, palmitic acid (16:0); PANSS, Positive and Negative Syndrome Scale; SA, stearic acid (18:0); SCoRS, Schizophrenia Cognition Rating Scale; SOFAS, Social and Occupational Functioning Assessment Scale.
a. Differences between groups were examined by qui-square test (gender) or Mann-Whitney U test (others) (* p < 0.05).
b. BACS composite score was calculated by averaging all z-scores of the six primary measures from the BACS.
c. Data are ranging from 0 to 10, with larger number representing more worse function.
d. Data are ranging from 0 to 100. Healthy subjects generally have a score ranging from 90 to 100.
e. n-6/n-3 ratio=AA/(EPA+DHA)
f. Omega3 index=EPA+DHA

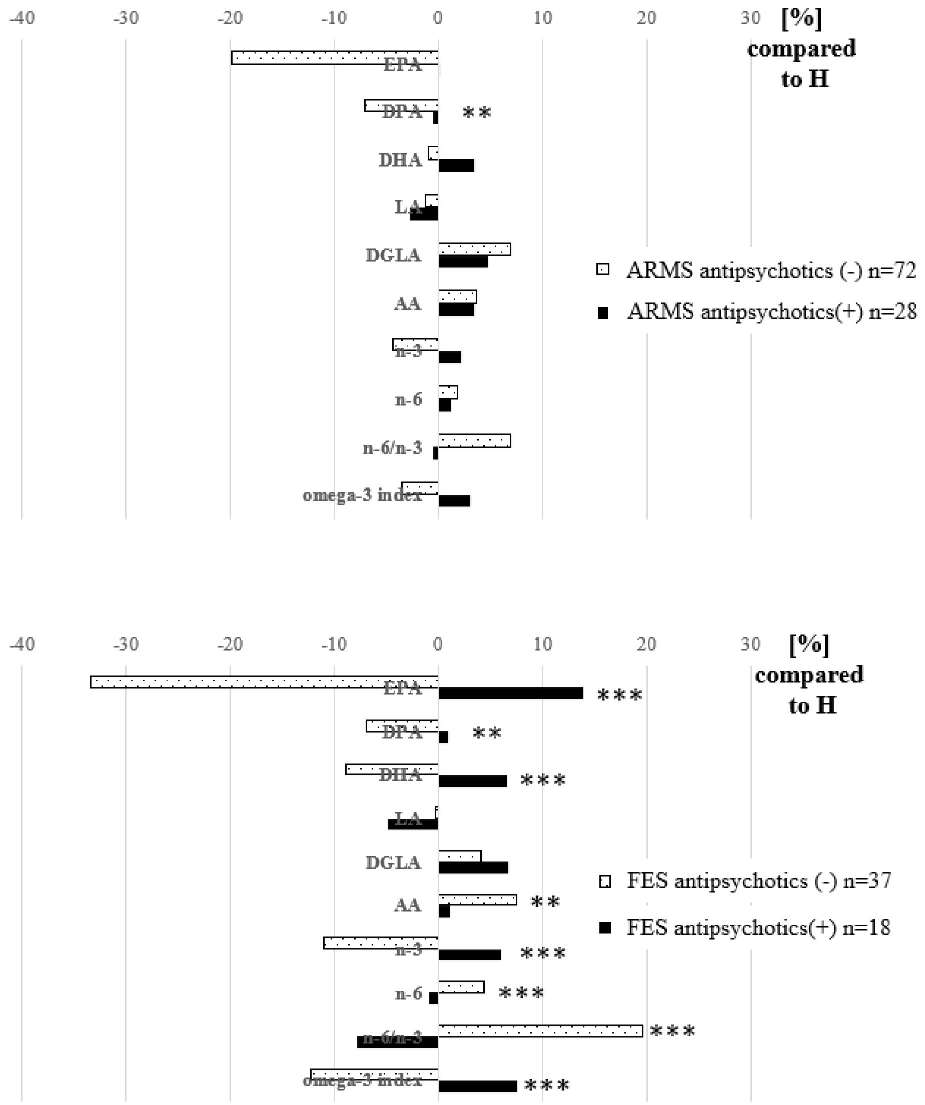

Suppl 5 Fatty acid levels for antipsychotic (+) and (-) groups compared to H group [%].

Note:
Upper and lower panels represent ARMS and FES, respectively.

Data was calculated by Mann-Whitney's U test.
***p<0.001, **p<0.01.

Abbreviate: AA, arachidonic acid (20:4 n-6); ARMS, at-risk mental state; DGLA, dihomogammalinolenic acid (20:3 n-6); DHA, docosahexaenoic acid (22:6 n-3); DPA, docosapentaenoic acid (22:5 n-3); EPA, eicosapentaenoic acid (20:5 n-3); FES, first episode schizophrenia; H, healthy control; LA, linoleic acid (18:2 n-6).

Suppl 6. Correlations between fatty acid levels and antipsychotic dosage.

		ARMS (n=93)		FES (n=55)	
		rho	p	rho	p
saturated	PA	0.04	0.68	0.12	0.38
	SA	-0.09	0.38	-0.15	0.28
n-9 monounsaturated	OA	-0.15	0.14	-0.36	0.008**
	NA	0.19	0.07	0.09	0.52
n-3 polyunsaturated	EPA	0.13	0.23	0.66	< 0.00001***
	DPA	0.27	0.009**	0.46	0.0004***
	DHA	0.09	0.39	0.52	0.00007***
n-6 polyunsaturated	LA	-0.04	0.69	-0.21	0.13
	DGLA	-0.04	0.68	0.02	0.91
	AA	0.02	0.85	-0.35	0.009**
summary value	n-3 total	0.13	0.22	0.61	< 0.00001***
	n-6 total	-0.01	0.89	-0.48	0.0002***
	n-6/n-3 ratio[a]	-0.09	0.39	-0.61	< 0.00001
	Omega3 Index[b]	-0.05	0.61	-0.56	0.00001***

Note: Values are Spearman's rank correlation coefficient, calculated using semi-partial correlation analysis that only fatty acid indices were controlled by age as a covariate.

Abbreviate: AA, arachidonic acid (20:4 n-6); ARMS, at-risk mental state; DGLA, dihomogammalinolenic acid (20:3 n-6); DHA, docosahexaenoic acid (22:6 n-3); DPA, docosapentaenoic acid (22:5 n-3); EPA, eicosapentaenoic acid (20:5 n-3); FES, first episode schizophrenia; LA, linoleic acid (18:2 n-6); NA, nervonic acid (24:1 n-9); OA, oleic acid (18:1 n-9); PA, palmitic acid (16:0); SA, stearic acid (18:0).

a. n-6/n-3 ratio=AA/(EPA+DHA)

b. Omega3 index=EPA+DHA

***p<0.001, **p<0.01, *p<0.05.

Suppl 7. Correlations between fatty acid levels and duration of illness in first episode schizophrenia.

		FES (n=18)	
		rho	p
saturated	PA	0.10	0.70
	SA	-0.24	0.36
n-9 monounsaturated	OA	-0.05	0.86
	NA	0.44	0.08
n-3 polyunsaturated	EPA	-0.16	0.55
	DPA	-0.12	0.65
	DHA	0.27	0.30
n-6 polyunsaturated	LA	-0.13	0.61
	DGLA	-0.12	0.65
	AA	0.07	0.80
summary value	n-3 total	0.15	0.57
	n-6 total	0.15	0.57
	n-6/n-3 ratio[a]	-0.14	0.60
	Omega3 Index[b]	-0.13	0.61

Note: Values are Spearman's rank correlation coefficient, calculated using semi-partial correlation analysis that only fatty acid indices were controlled by age as a covariate.

Abbreviate: AA, arachidonic acid (20:4 n-6); DGLA, dihomogammalinolenic acid (20:3 n-6); DHA, docosahexaenoic acid (22:6 n-3); DPA, docosapentaenoic acid (22:5 n-3); EPA, eicosapentaenoic acid (20:5 n-3); FES, first episode schizophrenia; LA, linoleic acid (18:2 n-6); NA, nervonic acid (24:1 n-9); OA, oleic acid (18:1 n-9); PA, palmitic acid (16:0); SA, stearic acid (18:0).

a. n-6/n-3 ratio=AA/(EPA+DHA)

b. Omega3 index=EPA+DHA

***p<0.001, **p<0.01, *p<0.05.
